# Structural design and effect analysis on a new type of hydraulic oscillator driven with double valve groups

**DOI:** 10.1038/s41598-022-20116-8

**Published:** 2022-09-20

**Authors:** Hou Lingxia, Sun Qiaolei, Deng Long, Liu Yuwei, Feng Ding

**Affiliations:** 1 School of Mechanical Engineering, Yangtze University, Jingzhou, 434023 Hubei China; 2 Hubei Engineering Research Center for Oil and Gas Drilling and Completion Tools, Jingzhou, 434023 Hubei China

**Keywords:** Mechanical engineering, Energy storage

## Abstract

A new hydraulic oscillator was designed, which was able to adjust pressure fluctuations through two sets of dynamic and fixed valves. Hydraulic oscillators can meet the frequency and axial force requirements of drilling at lower drilling fluid flow than general hydraulic oscillators. Oscillator structure was described in detail and over-flow area between the two sets of dynamic and fixed valves was calculated. Based on drilling fluid flow difference, the influence of different fluid flows on oscillator pressure drops was analyzed, its influence law was determined by the finite element software Fluent, and its effect was verified by numerical simulations. The related research is of great significance in the selection of dynamic and fixed valves and provides the theoretical basis for the optimization of the structural parameters of double-valve hydraulic oscillators.

## Introduction

Static friction between drilling tool and borehole wall is increased with the increase of well depth and horizontal section in conventional drilling processes^1,2^, which seriously affects drill bit rock, drilling tool life, and drilling speed and even causes downhole accidents such as sticking, drilling, etc.^3–5^. Many research works have been conducted around the world on reducing the frictional resistance of drilling^6–8^ and hydraulic oscillators are one of the most interesting technologies^9,10^.

The hydraulic action of hydraulic oscillators causes axial vibration in drilling assembly, which can transform the static friction of drilling assembly into dynamic friction. In this way, the backing pressure of horizontal well can be effectively solved and drilling speed was improved. Zhang et al.^11^ introduced a self-excited hydraulic oscillator and experimentally analyzed its frequency and amplitude characteristics. Xu^12^ and Liu et al.^13^ evaluated the effect of self-oscillating rotary percussion drilling tools in the field. Liu et al.^14^ designed a new type of hydraulic oscillator with axial vibration induced by hydraulic pulse and experimentally described its structure and principle. Li et al.^15^ developed a hydraulic oscillator for φ215 mm borehole and carried out indoor simulations and field experiments. Wang et al.^16^ designed a hydraulic oscillator and investigated its material and structure. The effect of hydraulic oscillator is demonstrated by thrust load, pressure frequency test and field application.

According to the numerical solutions of the Navier–Stokes equations from Fluent modeling and experimental in the system “Pipeline Fittings”, Karpenko et al.^17^ analyzed the influence of hydrodynamic processes on the development of the turbulent flow of a fluid. Yu et al.^18^ designed a hydraulic oscillator and analyzed its key mechanical parameters, changing law and vibration characteristics. Richard^19^, Liu^20^, Leus^21^, Wicks^22^ were proposed different models of axial vibrations for hydraulic oscillator analysis.

However, in order to achieve the requirements of oscillation force, frequency, etc., most of the studied hydraulic oscillators required more than 30 L/s drilling fluid^23,24^; therefore, large flow drilling pump or additional drilling pumps were needed. Hence, we designed a hydraulic oscillator driven with double valve group suitable for drilling fluid flows of less than 30 L/s.

## Design of a hydraulic oscillator driven with double valve group

### The structure of hydraulic oscillator

**Figure 1:**
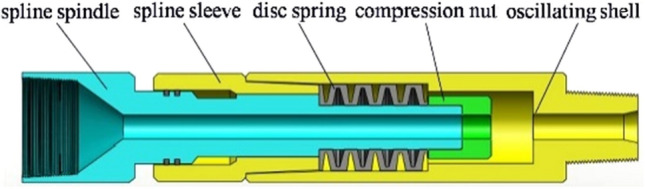
The oscillation section of oscillator.

**Figure 2:**
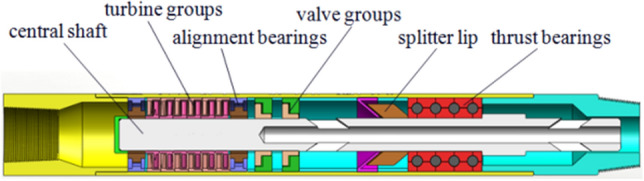
The drive section of oscillator.

The core of a hydraulic oscillator driven with double valve group consists of two parts, i.e., the upper section of oscillator and the lower end of drive section. As shown in Fig. 1, oscillation section was mainly composed of spline spindle, spline sleeve, disc spring, compression nut, oscillating shell, etc. Also, its main function was to convert pressure wave coming from the lower end to oscillating axial force of vibrating shell through disc spring. As can be seen in Fig. 2, drive section was mainly consisted of central shaft, turbine group, alignment bearings, dynamic valve, splitter lip, and thrust bearings. Oscillation section was connected with drive section through the screw thread. The main function of drive section was rotating center shaft by a turbine set to modulate periodic harmonic waves through periodically changing flow channel by the rotation of dynamic valves.

### Working principle

**Figure 3:**
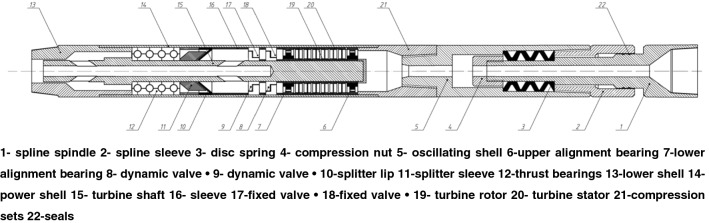
The structure of hydraulic oscillator driven with double valve groups.

Figure 3 shows the overall structure of hydraulic oscillators driven with double valve groups. Oscillator spline spindle top connected upper drill string. The lower shell of oscillator connected lower drill pipe. Oscillator power was supplied from drilling fluid, which transformed the liquid energy of drilling fluid into high-speed mechanical energy through turbine stator and rotor and the rotation of central shaft in drive section was driven by turbine rotor. The rotation of central shaft rotated e dynamic valves and over-flow area between dynamic and fixed valves was periodically changed during rotation. The maximum pressure drop was observed for minimum over-flow area and minimum pressure drop was witnessed for maximum over-flow area. The resulting pressure wave acted the compression nut in oscillation section; therefore, different pressures between the upper and lower end faces of compression nut generated an axial force. Axial force caused butterfly spring to continuously compress and recover to save and release energy. This made oscillating joint axially reciprocated and drill tool generated high frequency axial creep.

### Structural advantages

1. Turbine drive section was consisted of a pure metal component, which had some advantages such as high temperature resistance, strong erosion resistance, long service life and high efficiency over screw-driven hydraulic oscillators.
2. Double-valve groups with double-flow change traditional single channel by splitter lip, pressure wave was modulated by two sets of valve groups, and pressure fluctuation was changed by adjusting angle and distance between fixed valves. Different pressure amplitudes and appropriate oscillation forces could be generated under the condition of constant flow rate, which made it easier to meet field requirements with lower liquid flow rates.
3. Reasonably flow channels of the valves. The structure of valve plate was optimized through actual operating conditions of the oscillator and flow channel characteristics. It can split the impurity in the drilling fluid by a certain flow area of the export channel which in the left of the valve port when the rotor rotates to a fully closed state.

## Analysis of the key structural parameters of oscillators

### Calculation of the flow area of valve assembly

**Figure 4:**
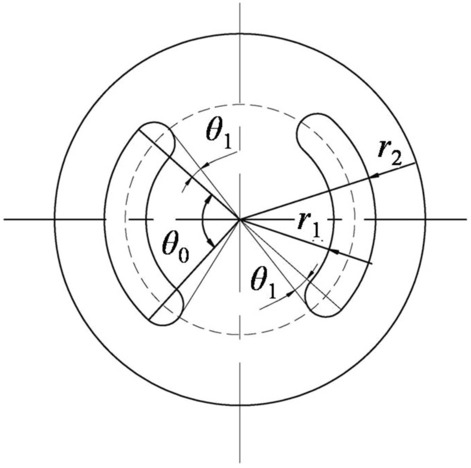
The structures of dynamic and fixed valves.

**Figure 5:**
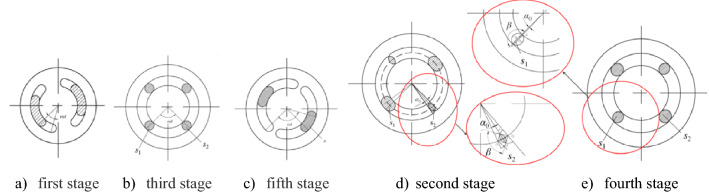
The variation of flow channels in one cycle.

Analysis one valve group for the hydraulic oscillator since the structure of the double valve groups are the same, and the structures of dynamic and fixed valves are shown in Fig. 4. The flow channels of valves were central symmetry, with flow channel outer diameter $r_{2}$, flow channel inner diameter $r_{1}$, the angle of standard annular region $\theta_{0}$, and circle radii on both sides $r_{3} ,r_{3} = (r_{2} - r_{1} )/2$. The whole dynamic valve rotation process was divided into five stages based on the change of flow channels in one cycle (rotation radian of $\pi$), as shown in Fig. 5.

Rotation process was illustrated by taking the following assumptions: the left channel of the fixed valve as the initial position of calculation, dynamic and fixed valves coincided at initial position, and dynamic valve rotated at a constant speed in work process. The left channel front of the dynamic valve did not intersect with the right channel of the fixed valve in the first stage. The left channel front of the dynamic valve t intersected with the right channel of the fixed valve in the second stage, intersection area $s_{1}$ was greater than $\pi r_{3}^{2}$ and intersection area $s_{2}$ was less than $\pi r_{3}^{2}$. The left channel front of the dynamic valve intersected with the right channel of the fixed valve in the third stage and intersection areas $s_{1}$ and $s_{2}$ were greater than $\pi r_{3}^{2}$ in the third stage. However, in the fourth stage, intersection area $s_{1}$ was smaller than $\pi r_{3}^{2}$ and intersection area $s_{2}$ was greater than $\pi r_{3}^{2}$. The left channel end of the dynamic valve did not intersect with the left channel of the fixed valve in the fifth stage and intersection area was $s$.The intersect dynamic valve left channel completely coincident with the right channel of the fixed valve and the intersect of dynamic valve right channel totally coincided with the left channel of the fixed valve at the end of one cycle, while rotation radian of dynamic valve was $\pi$.

The angular velocity of dynamic valve was assumed to be $\omega$ and rotation time was $t$. Flow channels area between the valves was calculated as follows.

1 $$A = \left\{ \begin{gathered} 2\pi r_{3}^{2} + (\theta_{0} - \omega t)(r_{2}^{2} - r_{1}^{2} ){\kern 1pt} \quad \left( {0 < t \le \frac{{\pi - \theta_{0} - 2\theta_{1} }}{\omega }} \right) \hfill \\ 2\pi r_{3}^{2} + (\theta_{0} - \omega t)(r_{2}^{2} - r_{1}^{2} ){\kern 1pt} {\kern 1pt} + 2\beta r_{3}^{2} - 4r_{0} \sin \left( {\frac{{\pi - \theta_{0} - \omega t}}{2}} \right) \times \sqrt {r_{3}^{2} - r_{0}^{2} \sin^{2} \left( {\frac{{\pi - \theta_{0} - \omega t}}{2}} \right)} \quad \left( {\frac{{\pi - \theta_{0} - 2\theta_{1} }}{\omega } < t \le \frac{{\pi - \theta_{0} }}{\omega }} \right){\kern 1pt} \hfill \\ 2\pi r_{3}^{2} + (2\theta_{0} - \pi )(r_{2}^{2} - r_{1}^{2} )\quad \left( {\frac{{\pi - \theta_{0} }}{\omega } < t \le \frac{{\theta_{0} }}{\omega }} \right) \hfill \\ 2\pi r_{3}^{2} + (\omega t - \pi + \theta_{0} )(r_{2}^{2} - r_{1}^{2} ) + 2\beta r_{3}^{2} {\kern 1pt} {\kern 1pt} - \left[ {4r_{0} \sin \left( {\frac{{\omega t - \theta_{0} }}{2}} \right)\sqrt {r_{3}^{2} - r_{0}^{2} \sin^{2} \left( {\frac{{\omega t - \theta_{0} }}{2}} \right)} } \right]\left( {\frac{{\theta_{0} }}{\omega } < t \le \frac{{\theta_{0} + 2\theta_{1} }}{\omega }} \right) \hfill \\ 2\pi r_{3}^{2} + (\omega t - \pi + \theta_{0} )(r_{2}^{2} - r_{1}^{2} )\quad \left( {\frac{{\theta_{0} + 2\theta_{1} }}{\omega } < t \le \frac{\pi }{\omega }} \right) \hfill \\ \end{gathered} \right.$$

where $\theta_{1}$ is the angle between tangent line which is the origin point to the sides circle of the valve and the line which is the origin point to the center of the sides circle, $\theta_{1} = {\text{arcsin}}\frac{{r_{3} }}{{r_{0} }}$,where $r_{0}$ is the radius of flow channel pitch circle, $r_{0} = (r_{2} + r_{1} )/2$. $\beta$ is the angle between the two radii which are the center of the side circle to the intersection points of the dynamic and fixed valves.$\beta$ in the second and fourth stages could be calculated as follows.

2 $$\beta = \left\{ {\begin{array}{*{20}c} {2{\text{arccos}}\left[ {\frac{{(r_{2} + r_{1} )\sin \left( {\frac{{\pi - \theta_{0} - \omega t}}{2}} \right)}}{{r_{2} - r_{1} }}} \right]{\kern 1pt} } & {\quad \left( {\frac{{\pi - \theta_{0} - 2\theta_{1} }}{\omega } < t \le \frac{{\pi - \theta_{0} }}{\omega }} \right)} \\ {2{\text{arccos}}\left[ {\frac{{(r_{2} + r_{1} )\sin \left( {\frac{{\omega t - \pi + \theta_{0} }}{2}} \right)}}{{r_{2} - r_{1} }}} \right]{\kern 1pt} } & {\quad \left( {\frac{{\theta_{0} }}{\omega } < t \le \frac{{\theta_{0} + 2\theta_{1} }}{\omega }} \right)} \\ \end{array} } \right.$$

Flow channel areas had to change all the time depending on field application requirements which could ensure that pressure drop between valve plates could be continuously changed so that disc spring could produce periodic changes. Therefore, the shorter the third stage, the better. Finally, this tool was designed for $\theta_{0} = \pi /2$ to make sure that the time of the third stage was 0 and flow channel areas could be simplified as follows.

3 $$A = \left\{ \begin{gathered} 2\pi r_{3}^{2} + (\pi /2 - \omega t)(r_{2}^{2} - r_{1}^{2} )\quad \left( {0 < t \le \frac{{\pi - 4\theta_{1} }}{2\omega }} \right) \hfill \\ 2\pi r_{3}^{2} + (\pi /2 - \omega t)(r_{2}^{2} - r_{1}^{2} ){\kern 1pt} + 2\beta r_{3}^{2} - 4r_{0} \sin \left( {\frac{\pi /2 - \omega t}{2}} \right) \times \sqrt {r_{3}^{2} - r_{0}^{2} \sin^{2} \left( {\frac{\pi /2 - \omega t}{2}} \right)} \quad \left( {\frac{{\pi /2 - 2\theta_{1} }}{\omega } < t \le \frac{\pi /2}{\omega }} \right) \hfill \\ 2\pi r_{3}^{2} + (\omega t - \pi /2)(r_{2}^{2} - r_{1}^{2} ) + 2\beta r_{3}^{2} - 4r_{0} \sin \left( {\frac{\omega t - \pi /2}{2}} \right)\sqrt {r_{3}^{2} - r_{0}^{2} \sin^{2} \left( {\frac{\omega t - \pi /2}{2}} \right)} \quad \left( {\frac{\pi /2}{\omega } < t \le \frac{{\pi /2 + 2\theta_{1} }}{\omega }} \right) \hfill \\ 2\pi r_{3}^{2} + (\omega t - \pi /2)(r_{2}^{2} - r_{1}^{2} )\quad \left( {\frac{{\pi /2 + 2\theta_{1} }}{\omega } < t \le \frac{\pi }{\omega }} \right) \hfill \\ \end{gathered} \right.$$

### Influence of inlet flow rate on pressure drop in valve group

The axial forces and pressure fluctuations of double-valve driving hydraulic oscillators were mainly produced by the change of flow channel between dynamic and fixed valves. Since both valve groups produced pressure drops, pressure fluctuations in oscillator segments generated superposition effects enhancing their performance. One valve group was adopted for analysis and instantaneous pressure drops between dynamic and fixed valves followed thin hole theory.

4 $$Q = C_{d} A\sqrt {2\Delta p/\rho }$$

where $C_{d}$ is flow coefficient which is in the range of 0.6 to 0.8, $\rho$ is drilling fluid density (kg/m^3^), *Q* is flow drilling fluid (m^3^/s), A is flow channel area (m^2^), and $\Delta p$ is the pressure drops of valve group (Pa).

Equation (4) was as follows.

5 $$\Delta p = \frac{{\rho Q^{2} }}{{2C_{d}^{2} A^{2} }}$$

Equation (5) shows that the flow area of valve group could be controlled to change pressure drop by changing valve parameters in the design. Due to the limits of valve material, upper working pressure drop, working environment, etc., the maximum pressure fluctuation could not be too high. In order to meet actual pressure fluctuations, the maximum pressure drop $\Delta p_{\max }$ corresponded to the minimum flow area $A_{\min }$ and the maximum pressure drop $\Delta p_{{m{\text{in}}}}$ corresponded to the minimum flow area $A_{{{\text{max}}}}$; $A_{\max }$ and $A_{\min }$ could be calculated as follows.

6 $$\begin{aligned} A_{\min } & = \frac{Q}{{C_{d} }}\sqrt {\frac{\rho }{2}} \frac{1}{{\sqrt {\Delta p_{\max } } }} \\ A_{{m{\text{ax}}}} & = \frac{Q}{{C_{d} }}\sqrt {\frac{\rho }{2}} \frac{1}{{\sqrt {\Delta p_{{m{\text{in}}}} } }} \\ \end{aligned}$$

**Figure 6:**
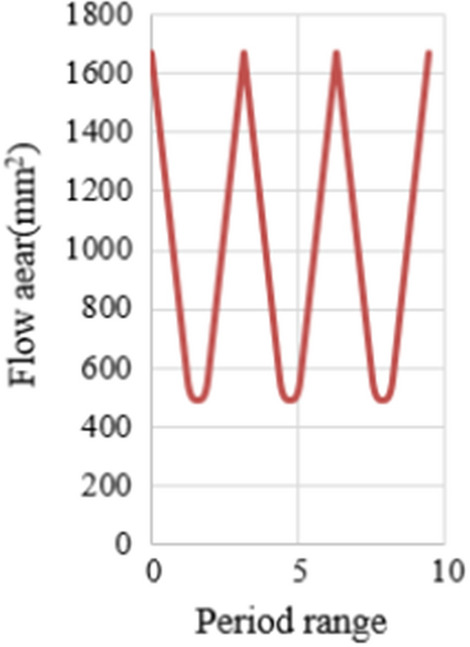
The change rules of flow area.

Field applications showed that the maximum hydraulic pressure consumption of hydraulic oscillator was supposed to be not greater than 4 MPa due to the restriction of underground space, relatively compact downhole tool structure, structure size limitation of rotary valve, etc.^25,26^. Therefore, the maximum pressure drop of one valve group was taken as 3.20 MPa considering turbine joint and local pressure loss. Combined with the external diameter of oscillator and the demand of maximum and minimum pressure drops of one valve group, the radii of valve plate were determined to be 70 mm, *r*_2_ = 42.5 mm,*r*_1_ = 30 mm, *r*_*0*_ = 36.25 mm, *r*_*3*_ = 3.25 mm, assuming $\rho = 1200\,{\text{kg}}/{\text{m}}^{3}$ and *C*_*d*_ = 0.8. Flow area was calculated by MATLAB software at each stage and the change rules of flow area is shown in Fig. 6. It could be calculated that $A_{\max } = 1669\,{\text{mm}}^{2}$ and $A_{{{\text{min}}}} = 490.88\,{\text{mm}}^{2}$.

**Figure 7:**
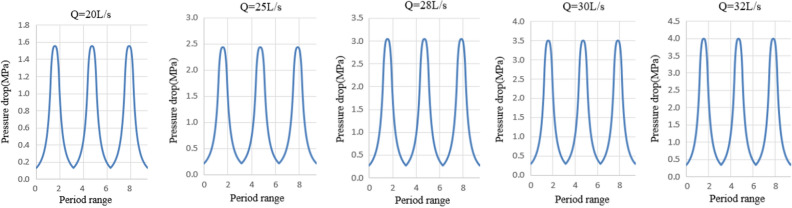
Relationship between pressure drop and flow rate.

Based on the analysis of the relationship between pressure drop and drilling fluid flow in hydraulic oscillators, the flow of drilling fluid to select field commonly used drilling fluid, flow respectively 20 L/s, 25 L/s, 28 L/s, 30 L/s, 32 L/s. Pressure drop change law with inlet flow was solved by MATLAB for two development of relative program, as shown in Fig. 7.

**Figure 8:**
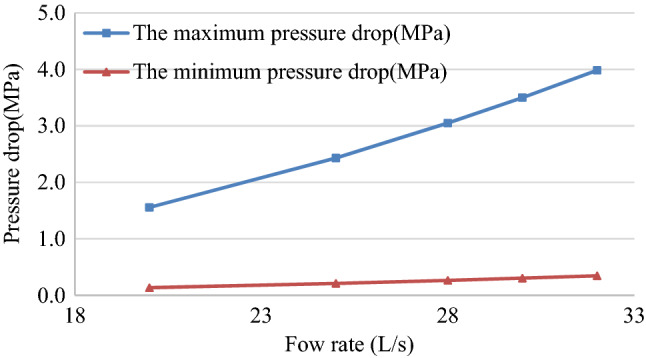
Relationship between maximum and minimum pressure drop with flow rate.

The variations of maximum and minimum pressure drops with flow rate are shown in Fig. 8.

The designed hydraulic oscillator produced continuous pressure fluctuations in a single cycle. The maximum pressure drop corresponding to the five flows were 1.56 MPa, 2.43 MPa, 3.05 MPa, 3.5 MPa and 3.98 MPa respectively. When flow rate was 28 L/s, the maximum pressure consumption of the valve group was 3.05 MPa and the average pressure drop was 1.25 MPa, which met design requirements. Meanwhile, flow could be easily realized by the pump group at the same time.

## Numerical simulation of hydraulic oscillator

In order to further study the performance of the design of double valve driving hydraulic oscillators, the work process of the fabricated hydraulic oscillator was simulated by the finite element software Fluent combined with the structure of hydraulic oscillator.

### Selection of turbulence model

Since the flow channel of double-valve driving hydraulic oscillator had multi-stage cross-section changes, fluid movement could be take the shape of rotary motion. Compared with standard k-turbulence model, RNG model takes into account rotation and swirl flows and it could better deal with high strain rate and flow of streamline^27^. Therefore, k-RNG turbulence model was selected for the numerical simulation of hydraulic oscillators.

### Establishment of finite element model

**Figure 9:**
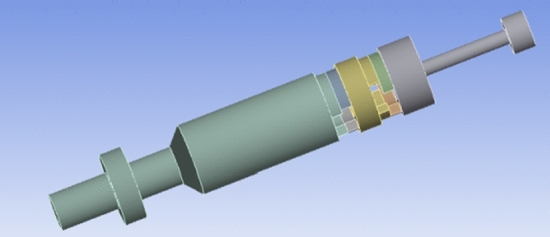
3D model of the flow channel of oscillator.

The 3D model of oscillator was built in SolidWorks software. For the sake of analysis before the analysis. The following basic assumptions were considered to simplify analysis, (1) numerical simulation mainly studied fluid pressure change in oscillating section; hence, the internal flow channel of oscillating section was fixed while modeling and spring effect was ignored. (2) To simplify the internal flow channel of drive section, turbine effect, righting bearing, thrust bearing and others were not taken into account. (3) Outlet of the lower part of the model of drive section was simplified. In addition, local refinement of flow channel was carried out and the final 3D model of flow channel is shown in Fig. 9.

**Figure 10:**
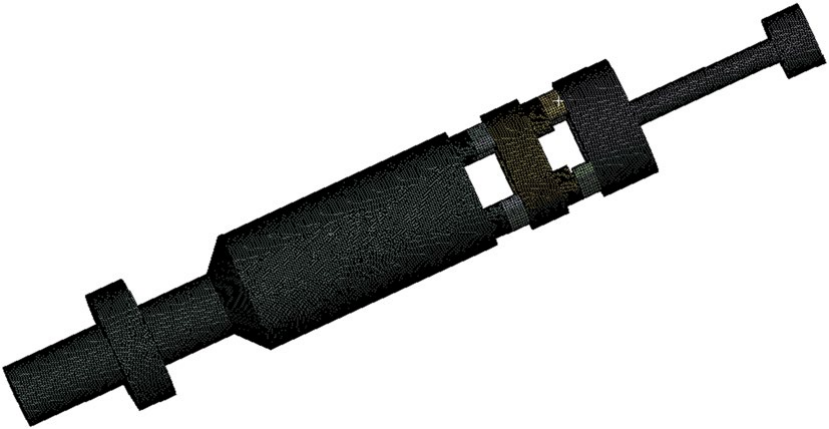
The mesh diagram of the 3D model.

The finished model was saved as an “xt” file and imported to Fluent of Workbench to divide the grid with node number of 504,777 and grid cell number of 2,505,046, as shown in Fig. 10.

After the completion of the mesh, the flow rate of drilling fluid (simulated with water) had to be translated into inlet speed to set the inlet boundary conditions combined with the actual operating conditions of the hydraulic oscillator. 20 cycles were considered and 25 data points were taken in each cycle. Based on theoretical design, dynamic valve speed was 8 r/s, outlet was set to free export, and the remaining boundary conditions were set in turn according to the actual parameters to complete the simulation.

### Analysis results

**Figure 11:**
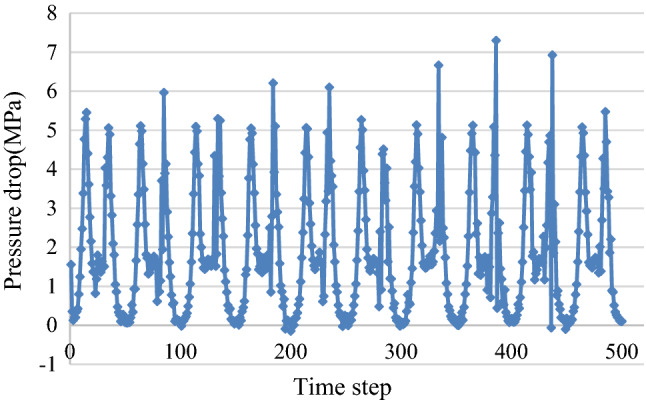
Variation law of pressure drop with time step.

Outlet pressure, oscillation front end, first valve group front end, and second valve group back end were obtained. Oscillator pressure drop was defined as the difference in the pressures of first valve group front end and second valve group back end, as shown in Fig. 11. Theoretically, the simulated pressure drop was two times as high as that theoretically calculated with one single valve group. Simulation results showed that maximum and average pressure drops were 7.30 MPa and 1.81 MPa in 20 cycles of simulation, respectively. The highest maximum pressure drop in one cycle was two times the theoretical value with 6.10 MPa and the basic simulation law was almost consistent with theoretical calculation.

## Summary and conclusion

1. A new type of turbo-driven hydraulic oscillator was designed in which pressure fluctuations were generated by double valve groups and oscillation force demand could be generated at lower flow rates mainly. Based on the principle of pressure wave superposition, the maximum pressure drops of the new type of turbo-driven hydraulic oscillator were 7.30 MPa in 20 cycles of simulation, which was two times the theoretical value with only one valve. The main structure of the designed oscillator was mad of pure metal components and its advantages include high temperature resistance, high erosion resistance, long service life and high efficiency.
2. The whole rotation process of hydraulic oscillator was divided into five stages during dynamic valve rotation based on flow channel changes in one cycle. The calculation model of valve flow area in five stages was established according to the geometrical model of each stage. The structural parameters of double valve group were designed based on the calculation model. Finally, on the basis of related parameters, the influences of different drilling fluid flows on the pressure drops of hydraulic oscillator were analyzed.
3. According to the pressure drops of hydraulic oscillator solved by MATLAB, the change law of the pressure drop under different drilling fluids was basically the same. While the flow rate from 20 to 32 L/s, the maximum pressure drop was approximately linear with the flow rate, the minimum pressure drop value to little change, and when flow rate was 28 L/s, the designed requirements was met.
4. The finite element software Fluent was applied to simulate the hydraulic oscillator by establishing a 3D flow channel. Based on the principle of pressure wave superposition, the maximum pressure drops of the new type of turbo-driven hydraulic oscillator were 7.30 MPa in 20 cycles of simulation, and the results showed that pressure drop in double valve groups was two times that of a single valve group, which indicated the feasibility of turbo-driven hydraulic oscillators.

## Data Availability

The datasets used and/or analysed during the current study available from the corresponding author on reasonable request.
